# Development of a Core Body Thermometer Applicable for High-Temperature Environment Based on the Zero-Heat-Flux Method

**DOI:** 10.3390/s23041970

**Published:** 2023-02-09

**Authors:** Hanzi Lu, Shun Aratake, Hisashi Naito, Masamichi Nogawa, Tetsu Nemoto, Tatsuo Togawa, Shinobu Tanaka

**Affiliations:** 1 Graduate School of Natural Science & Technology, Kanazawa University, Kanazawa 920-1164, Japan; 2 Institute of Science and Engineering, Kanazawa University, Kanazawa 920-1164, Japan; 3 Faculty of Health Sciences, Komatsu University, Komatsu 923-0961, Japan; 4 Institute of Medical, Pharmaceutical and Health Sciences, Kanazawa University, Kanazawa 920-0942, Japan; 5 Advanced Research Center for Human Sciences, Waseda University, Tokorozawa 359-1192, Japan

**Keywords:** core body temperature, zero-heat-flux method, hot environment solution, core body thermometer, Peltier module

## Abstract

Monitoring core body temperature (CBT) allows observation of heat stress and thermal comfort in various environments. By introducing a Peltier element, we improved the zero-heat-flux core body thermometer for hot environments. In this study, we performed a theoretical analysis, designed a prototype probe, and evaluated its performance through simulator experiments with human subjects. The finite element analysis shows that our design can reduce the influence of external temperature variations by as much as 1%. In the simulator experiment, the prototype probe could measure deep temperatures within an error of less than 0.1 °C, regardless of outside temperature change. In the ergometer experiment with four subjects, the average difference between the prototype probe and a commercial zero-heat-flux probe was +0.1 °C, with a 95% LOA of −0.23 °C to +0.21 °C. In the dome sauna test, the results measured in six of the seven subjects exhibited the same trend as the reference temperature. These results show that the newly developed probe with the Peltier module can measure CBT accurately, even when the ambient temperature is higher than CBT up to 42 °C.

## 1. Introduction

Core body temperature (CBT), compared with skin temperature, which is easily affected by external temperature, is a key indicator of fever, thermal strain [1], and personal thermal comfort in a high-temperature environment. Fever-prone patients [2,3] and the population at risk of heatstroke (including athletes [4,5], outdoor workers [6], and firefighters [7]) require non-invasive CBT thermometers for health management and monitoring of thermal strain. This study aims to develop a non-invasive CBT thermometer with reasonable accuracy that is available at wider ambient temperature range.

Conventional direct measurements of CBT involve probes inserted into body orifices, such as the rectum or esophagus, for accurate measurements; however, it is difficult for the subject to tolerate prolonged measurements while awake [8]. As an alternative, infrared scanning thermometers can quickly measure the tympanic temperature within a few seconds. However, some studies [9,10,11] have claimed that its accuracy remains questionable, owing to the complex structure of the auditory canal. Ingestible thermometry capsules were used to measure the temperature of the gastrointestinal tract from the inside of the body [12], but their position in the body is uncontrollable, and the result may be easily influenced by the intake of food or fluid [13,14]. Patch-type temperature probes [15] and wrist-type thermometers [16] have been developed. However, the effectiveness of these probes for actual human measurements and their performance in high-temperature environments have not been thoroughly investigated.

The zero-heat-flux (ZHF) method was developed by Fox et al. as a non-invasive method for continuously measuring CBT [17]. Its measurement accuracy was improved by Togawa et al. [18], and it has been widely used for perioperative temperature management. In this method, the surface of the skin is covered by a heat insulator with a heater to compensate for outward heat dissipation. The thermistors were placed above and below the insulator layer to measure the heater temperature (T_Heater_) and skin temperature (T_Skin_), respectively. The heater was then controlled to maintain T_Heater_ at T_Skin_. When the state of thermal equilibrium is maintained, CBT can be measured indirectly and continuously from the skin surface.

Clinical applications of ZHF thermometers have been attempted in postoperative care [19,20] and neonatal care [21]. The commercial ZHF thermometer model delivered by Terumo Co. has been widely used, especially in intensive care after open-heart surgery.

The ZHF method has been proven to have excellent clinical performance [22]; however, it cannot operate in a high-temperature environment. The heater works only when T_Heater_ is lower than T_Skin_, which means that when the ambient temperature of the probe is higher than the CBT, the heater is turned off. In such a situation, the temperature gradient from the outside to the inside results in significant measurement errors, limiting the use of this method to only stable clinical settings [23].

However, our co-author (T.T.) demonstrated that a ZHF probe could maintain heat equilibrium and measure in a high-temperature environment if a Peltier module and radiator were placed instead of a heater [24]. In this study, we redesigned and constructed a ZHF probe with a Peltier module and insulator, investigated its behavior in detail, and we discuss its performance and wearability in practical applications.

## 2. Materials and Methods

### 2.1. Outline of the Structure of the Prototype Probe

**Figure 1:**
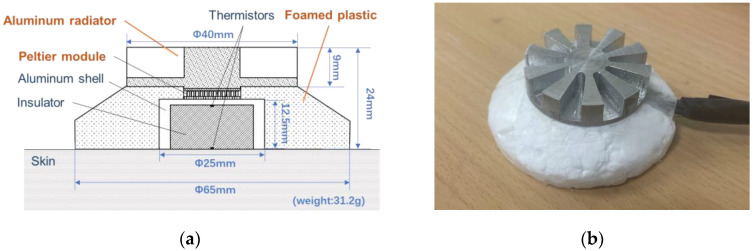
(**a**) Cross-section of the prototype probe and (**b**) external appearance of the probe.

On the basis of the results of the aforementioned study [19], a prototype zero-heat-flux probe using a Peltier element was developed, as shown in Figure 1. The left figure shows the cross-section of the prototype probe, which consists of a radiator, Peltier module (NL1023T-03AC, Marlow Industries, Inc., Dallas, TX, USA), thermistors (PSB-S7, SHIBAURA Electronics Co., Ltd., Saitama, Japan), aluminum shell, and insulation material (foamed plastic). According to our previous study [25], the peripheral skin surface around the aluminum shell is covered by an insulator to reduce heat dissipation from the skin near the shell, thus reducing the power consumption and improving the wearability of the probe. The right side of the figure shows the appearance of the prototype probe, which weighs 31.2 g.

**Figure 2:**
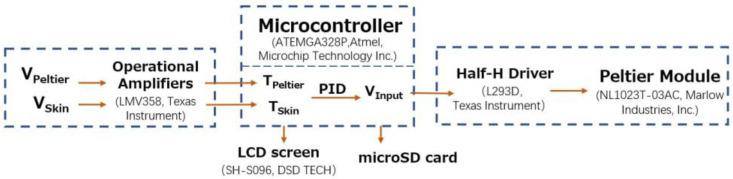
Block diagram of the control system.

A block diagram of the control system is shown in Figure 2. The operation of the control system involved controlling the current drain of the Peltier module to maintain the Peltier side temperature at the skin surface. The potential differences between the thermistors on the skin side and Peltier side (V_Skin_ and V_Peltier_) were amplified by operational amplifiers and sent to the microcontroller unit (MCU) (ATmega328P, Atmel, Microchip Technology Inc., Chandler, AZ, USA). The MCU reads the voltages and uses a correction formula to obtain the temperatures on the skin and Peltier element sides (T_Skin_ and T_Peltier_). On the basis of these two temperatures, the MCU uses a proportional–integral–derivative (PID) algorithm to obtain the voltage added to the Peltier element (V_Input_) and passes it to the half-H driver. The half-H driver (L293D, Texas Instruments) is capable of supplying high voltage and high current (max. 36 V, 600 mA). Polarity reversal was used to power the Peltier modules.

The servo control loop gains of the proportional, integral, and differential operations (K_p_, K_i_, and K_d_) are determined on the basis of the finite element analysis results described below. When the outside temperature was lower than the skin temperature, the heat flux was compensated for by heating the body surface, as in conventional core body thermometers. When the ambient temperature was higher than the skin temperature, the heat flux could still be compensated for by changing the current direction, cooling the body surface, and maintaining regular measurements.

For data collection, the results (T_Skin_, T_Peltier_, and V_Input_) were recorded using a 16 GB micro-SD card and displayed on a 0.9-inch LCD screen (SH-S096, DSD TECH).

### 2.2. Finite Element Analysis

Before the fabrication of the probe, we performed a finite element model analysis to verify whether our design could compensate for the heat flux and accurately detect the CBT at different external temperatures. We built a 3D model based on COMSOL Multiphysics LiveLink for SOLIDWORKS^®^.

In this 3D model, the prototype probe was placed on a thermally homogeneous cylindrical skin tissue with a radius of 80 mm and thickness of 10 mm. Each probe component is assumed to be a thermally homogeneous medium, and the deep tissue under the skin is assumed to be a constant-temperature medium at the core temperature. The physical property parameters listed in Table 1 were obtained from the COMSOL v5.6 material library.

For the boundary conditions, the contact surfaces of the skin and probe with outside air were set up for natural thermal convection. The convection heat transfer coefficient (free convection and gas) was set to 5 W/(m^2^·K) for consistency with the skin (Table 2), ignoring the effect of probe geometry on this coefficient [26]. In addition, the lateral borders of the skin were thermally insulated.

For the initial condition setting, the bottom surface of the skin was set to a core body temperature (37 °C), and the initial skin temperature condition was a uniform distribution of skin at the ambient temperature under the same natural-convection conditions. The initial temperature of the entire probe was set to 25 °C.

As shown in Table 2, we simulated the measurements the prototype probe could achieve after certain measurement durations as well as the time required to reach a steady-state measurement under two external temperature conditions (25/42 °C). During this process, the PID algorithm was used to calculate the input voltage (V_Input_) applied to the Peltier module. The PID parameters are listed in Table 2.

The duration of the simulated experiment was set to 3500 s, where the core body temperature (T_CBT_) was set to 37 °C for the first 2000 s. After obtaining steady-state temperature results, we increased T_CBT_ to 38 °C and observed whether the probe could capture the temperature changes gradually transmitted to the skin surface.

### 2.3. Simulator Experiments

**Figure 3:**
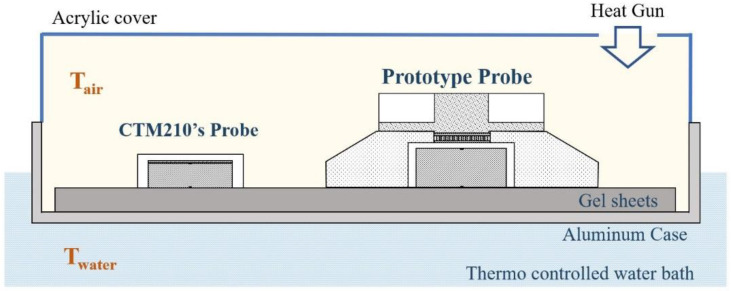
Cross-sectional view of the experimental setup.

The temperature measurement accuracy of the prototype probe was evaluated through a simulator experiment using a thermostatic water bath. As shown in Figure 3, an aluminum case was immersed in a thermostatic water bath (C-650, TAIYO TAITE C, room temperature~100 °C), and a gel sheet (HTCH2-150-150, MISUMI, thickness: 6 mm) was attached to the inner bottom of the bath. A prototype probe and a probe for a commercial core body thermometer (CoreTemp CTM-210, Terumo) were placed on the gel sheet. The temperature results of the prototype device (T_Proto_) were recorded every 1 s, whereas those of the commercial device T_CTM_ were recorded every 2 s. At the beginning of the experiment, the water temperature (T_water_) was set to 37 ± 0.5 °C. The T_water_ and air temperatures (T_air_) were monitored using a thermistor thermometer (3312A, INSTRULAB).

The experimental protocol began with a preparatory phase (Phase I), in which each probe was left open at room temperature for 40 min until the output stabilized. The high-temperature phase (Phase II) then followed, where an acrylic cover covered the entire case and a hot air gun was used to blow hot air at approximately 45 °C inward for 20 min. At the start of the recovery period (Phase III), the acrylic cover was removed so that the temperature returned to room temperature and was held at room temperature for 20 min.

### 2.4. Simultaneous Measurement of the Prototype and CTM-210 While Exercising at Room Temperature

The measurement accuracy and time delay of the prototype probe at room temperature were investigated through simultaneous measurements using a commercially available core body thermometer (CoreTemp CTM-210, Terumo) in a workload experiment using a bicycle ergometer.

Four healthy male adults (age, 22 ± 2 years) were enrolled in the experiment. The prototype and commercial probes were symmetrically fixed to the forehead using a belt. The experiments were performed at room temperature (25 ± 1 °C), and the procedure was as follows.

In Phase 1, the subject was at rest while seated for 30 min after fixation of the probe. In Phase 2, the subject performed 10 min of exercise (load intensity 75 W) on an ergometer (AEROBIKE 75XLIII, Konami Sports). In Phase 3, the subject was at rest while seated for another 20 min. The total duration of the experiment was 60 min. During this period, T_Proto_ and T_CTM_ were simultaneously recorded, as described above.

### 2.5. Simultaneous Measurement of the Prototype and CTM-210 in the Domed Sauna Experiment

The effects of changes in the ambient temperature on the measurement accuracy of the prototype and commercial probes were studied in the following manner.

Referring to the study by Sawatari et al. [27], a domed sauna (KMC DOME SAUNA Professional, Kobe Medi-care Co., Ltd., Kobe, Japan) was used to raise the external temperature of the subjects below their necks. Seven healthy male adults (23 ± 2 years old) were involved in the following experiment in the supine position, wearing shorts and T-shirts, with their upper abdomen exposed.

A prototype probe and CTM-210 probe were placed side-by-side near the midline of the upper abdomen, and the values of T_Proto_ and T_CTM_ were sampled at the aforementioned time points. Another CTM-210 probe was attached to the forehead area to simultaneously measure CBT in the head (T_Head_) as a reference value.

The ambient temperature T_Air_ was measured using a digital thermometer (CENTER 376, MK Scientific, Inc., Yokohama, Japan) with a dedicated sensor (Precision Pt100 Probe) fixed around the abdominal probe.

After each probe was secured, the subject was at rest in the supine position at room temperature (24 ± 1 °C) for 30–40 min (Phase A). In Phase B, a preheated domed sauna was placed over the subject’s body to raise their perimeter temperature from the feet to the neck up to 43 °C for 20 min. The sauna was then removed and the surrounding body temperature was allowed to return to room temperature for 20 min (Phase C).

The experiments were conducted with the approval of the Medical Ethics Committee of Kanazawa University (approval number: 2020-236 (087)).

## 3. Results

### 3.1. Finite Element Analysis

**Figure 4:**
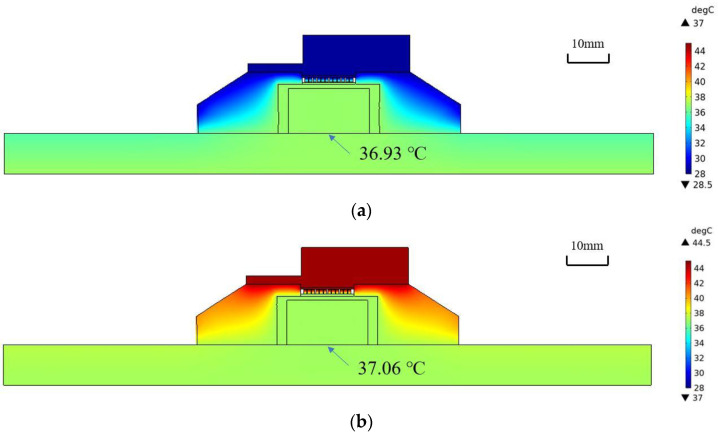
Results of finite element analysis. (**a**) T_Air_ = 25 °C, t = 1970 s; (**b**) T_Air_ = 42 °C, t = 1970 s.

The results of the finite element analysis are shown in Figure 4. The temperature distributions after 1970 s at T_Air_ = 25 °C and 42 °C are shown in (a) and (b), respectively. It can be observed that the skin temperature at the bottom center of the probe (36.93 °C and 37.06 °C) agrees well with the core body temperature (37 °C).

**Figure 5:**
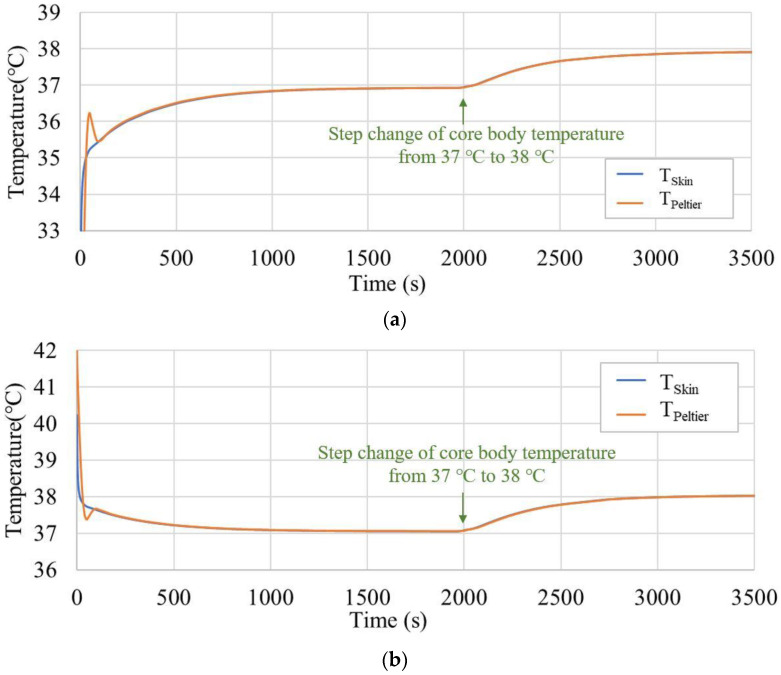
Thermistor temperature results from finite element analysis: (**a**) T_Air_ = 25 °C and (**b**) T_Air_ = 42 °C.

In Figure 5, T_Peltier_ shows signs of overshooting in the first two minutes but a quick realignment with T_Skin_ with PID regulation. Thereafter, both temperatures gradually approached the exact T_CBT_, and a thermal equilibrium state was obtained after approximately 1400 s at T_Air_ = 25 °C and approximately 800 s at T_Air_ = 42 °C, respectively. In response to the step change in deep temperature that occurred at 2000 s, T_Skin_ and T_Peltier_ first showed an exponential increase, after which they showed a first-order upward trend until a stable result was obtained. The errors of the measurements at 3500 s (37.91 °C and 38.03 °C) at different T_Air_ values (25 °C and 42 °C) were also within 0.1 °C.

The aforementioned finite element analysis results confirm that a thermal equilibrium state can be achieved when the ambient temperature is lower than the core body temperature. When the ambient temperature is higher than the core body temperature, accurate measurements can be performed using a Peltier module and a thermal insulation material.

We further analyzed the effects of the probe radius, thinness, and subcutaneous tissue thickness on the results. The results showed that the larger the thermal equilibrium radius of the probe, the larger the maximum depth that can be measured within the margin of error. Furthermore, the thinner the probe, the shorter the preparation time required to reach thermal equilibrium. It was also found that for thicker subcutaneous tissue or higher thermal conductivity of the same thickness, the maximum depth that can be measured within the margin of error is smaller.

### 3.2. Simulator Experiment

**Figure 6:**
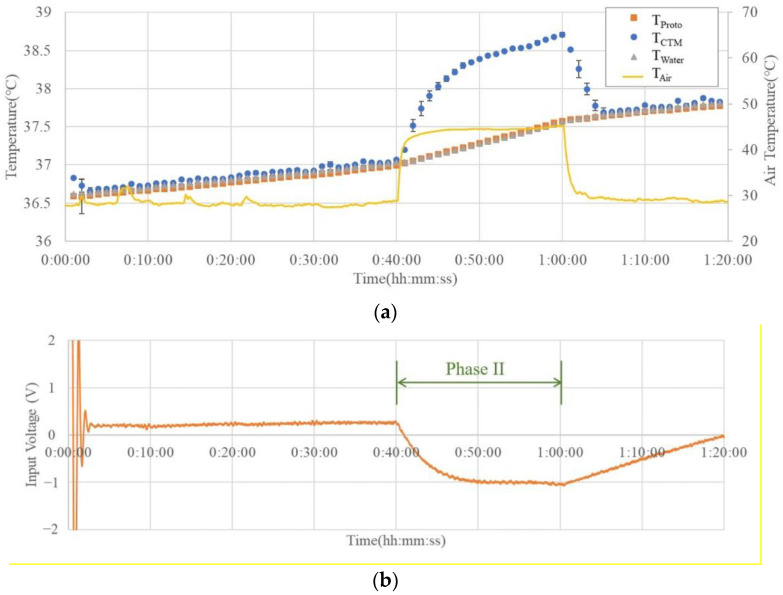
(**a**) Example of the results of simulator experiment showing the temperature change and (**b**) overtime change of Peltier module input voltage (V_Input_).

Figure 6 shows an example of the results of the simulator experiment. The temperature variation graph in (a) shows that the commercial probe matches the water temperature in Phases I and III. However, when the outside temperature increased during Phase II, the difference between the commercial probe measurement result (T_CTM_) and the actual water temperature (T_Water_) was greater than 1 °C at the maximum. A commercial probe cannot accurately measure the water temperature when the external temperature is higher than the water temperature.

On the other hand, the prototype probe showed a temperature difference of less than 0.02 ± 0.021 °C in all the phases, indicating that the water temperature could be measured with high accuracy, even in the high-temperature period of Phase II.

The graph in Figure 6b shows that the input voltage of the Peltier element (V_Input_) reverses its polarity and switches the module from heating to cooling when the external temperature exceeds the water temperature, thereby confirming that the designed control system operates effectively.

### 3.3. Simultaneous Measurement of the Prototype and CTM-210 in Ergometer Exercise at Room Temperature

**Figure 7:**
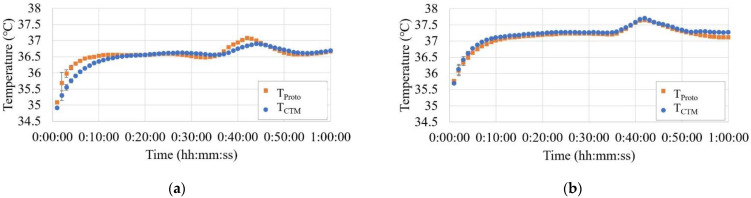

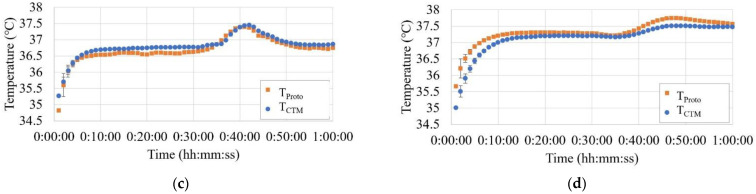
(**a**–**d**) Measurement results of the exercise experiment showing the core body temperature change of the four subjects (one minute average and SD).

Figure 7 shows the results of the core body temperature changes during the ergometer experiments. As shown in the figures, CBT increased by approximately 0.6 °C with the exercise load, and the prototype responded to this change almost identically to the commercial probe.

**Figure 8:**
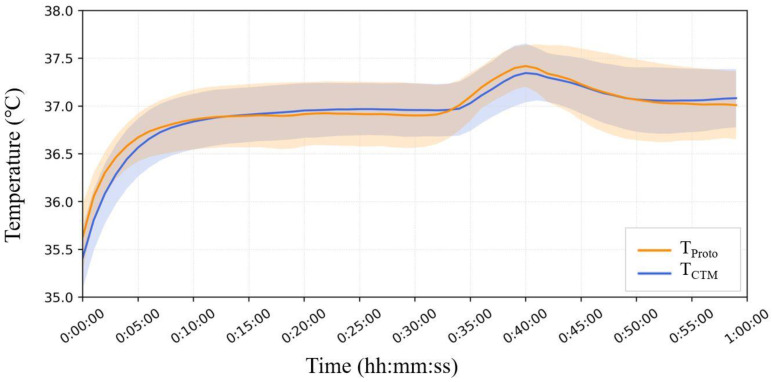
The means and error bands of the measurement results of all four subjects of the ergometer test.

Figure 8 shows the mean and error bands of the measurement results for all four subjects in the ergometer exercise experiment. Bland–Altman analysis of the results showed that the mean difference between T_Proto_ and T_CTM_ was −0.01 °C, and the 95% limits of agreement were −0.23 °C to +0.21 °C. The results of the two methods were in good agreement.

Cross-correlation analysis of the data from the four cases, focusing on the period of the shift in CBT induced by exercise load, showed a time lag in the narrow range of −1.5 min to 0.1 min with the CTM and an almost identical response.

### 3.4. Simultaneous Measurement of the Prototype and CTM-210 in the Domed Sauna Experiment

**Figure 9:**
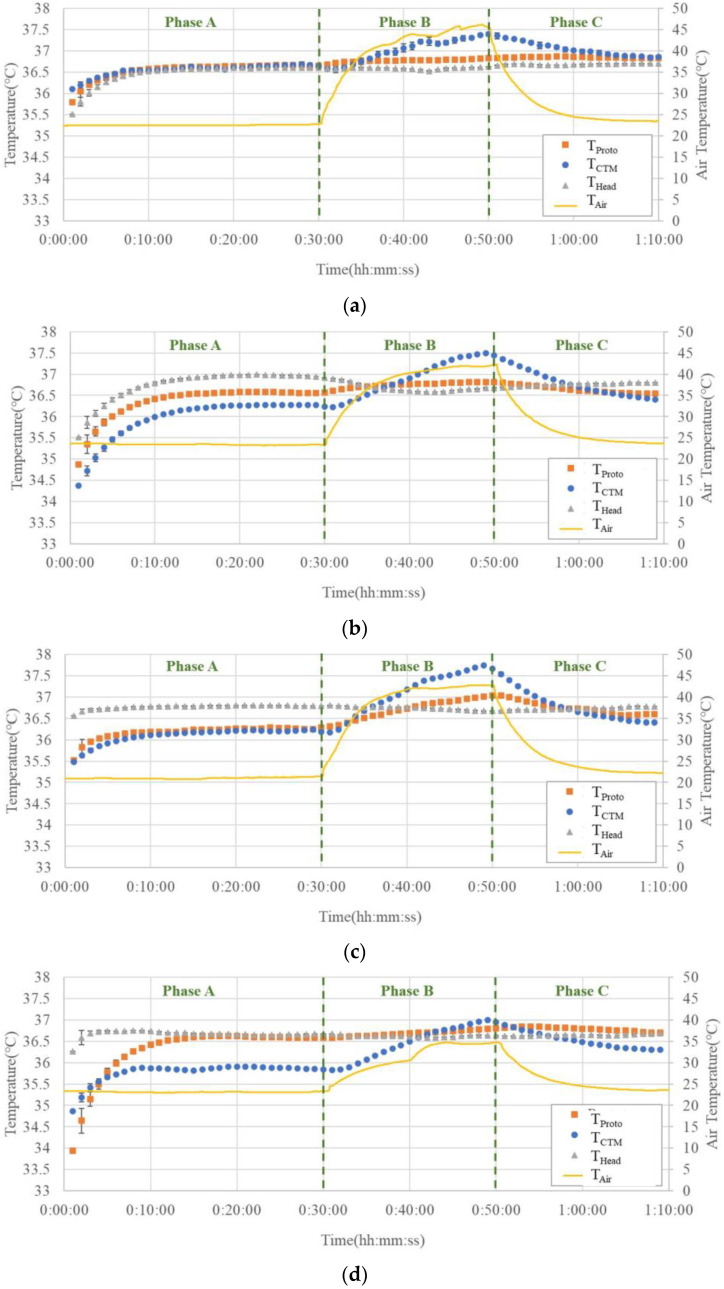

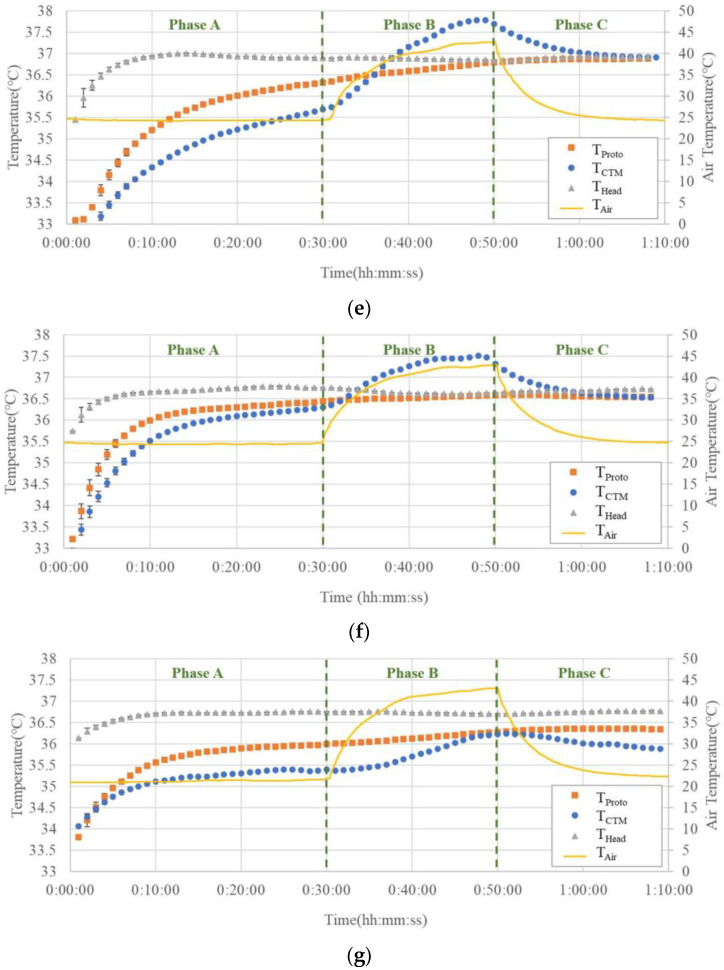
(**a**–**g**) Measurement results of the sauna experiments for seven subjects. T_Proto_ and T_CTM_ represent the abdominal deep temperatures measured by the prototype probe and CTM, respectively, and T_Head_ represents the forehead deep temperature measured by CTM before (Phase A), during (Phase B), and after (Phase C) hot air blowing.

Figure 9a–g show the temperature trends before, during, and after hot air blowing for all seven subjects.

In Phase A, T_Head_ tended to be greater than or equal to the temperature measured using both the abdominal probes. Although there were cases where T_CTM_ and T_Proto_ overlapped in the two abdominal probes, there were also cases where differences were observed, up to 0.5 °C.

In Phase B, T_Head_ did not change significantly, while T_CTM_ showed a significant increase, up to 1.3 °C. The results of T_Proto_ showed a small increase but were comparable to those of T_Head_.

In Phase C, T_CTM_ showed a significant decreasing trend with decreasing air temperature, whereas T_Head_ and T_Proto_ showed almost no change.

**Figure 10:**
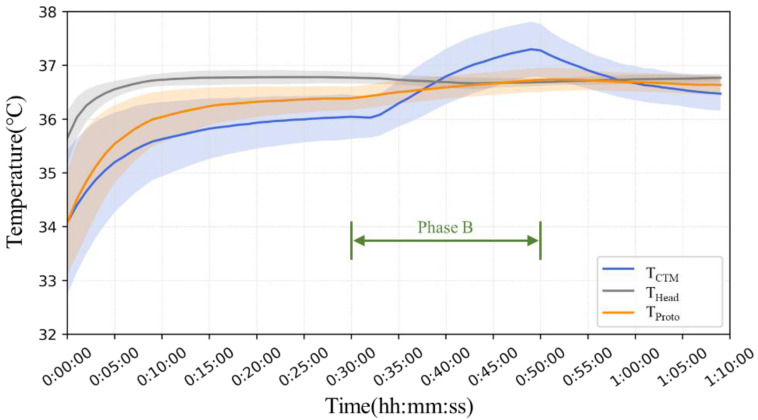
The means and error bands of the measurement results of all seven subjects.

Figure 10 shows the mean and standard deviation error bands for each minute for all seven subjects. In Phase A, T_Head_ was significantly greater than that measured on the abdomen, and T_CTM_ yielded lower results than T_Proto_. In Phase B, there was no significant change in T_Head_, while T_CTM_ showed a significant increase, up to 1.3 °C. The results of T_Proto_ showed a minor increase but were comparable to those of T_Head_. In the third phase, T_CTM_ showed a significant downward trend with air temperature, whereas T_Head_ and T_Proto_ showed almost no change.

**Figure 11:**
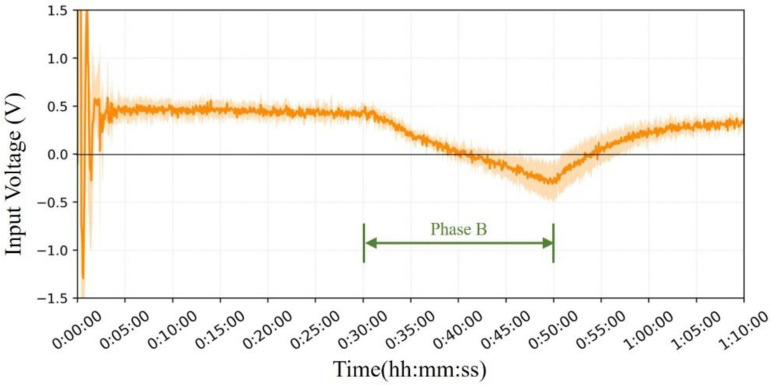
Average input voltage V_Input_ of the Peltier module with the error band for seven subjects.

Figure 11 shows the mean and error bands of the PID output under the influence of temperature. It can be observed that after the initial oscillation of the PID regulation, the V_Input_ stabilizes at approximately 0.5 V and continues to heat the probe interior to maintain the internal thermal equilibrium; in the second stage, the V_Input_ starts to gradually decrease and flips the current direction to start cooling the probe interior. After the external temperature returned to room temperature, the current gradually shifted back to the heating mode.

## 4. Discussion

### 4.1. Finite Element Analysis

Finite element analysis confirms that the probe with the Peltier element can measure the deep temperature in a hot environment. The focus was on the measurements at the end of temperature equilibration and at the initial time before reaching equilibrium. Our design accomplished deep temperature measurements in this simulation, kept the error within 0.1 °C, and successfully captured the deep temperature variation over a wide range of external temperatures (from 25 °C to 42 °C). After a step change in the core body temperature at 2000 s, the skin temperature exhibited a nearly exponential rise; thus, the system could be approximated as a first-order system regardless of the environmental temperature.

This result showed that the configuration and servocontrol systems of the model employed in this analysis are sufficient to satisfy the accuracy requirement of the clinical thermometer, in which measurement error should be less than 0.1 °C in the range of ordinary variation of body temperature.

In this analysis, we found that the thickness of the body–shell tissue at the measurement site affects the measurement results and preparation time. Reducing the thickness of the tissue and increasing the thermal conductivity may reduce temperature measurement errors and shorten the preparation time required for thermal equilibration. Therefore, it is recommended to use the forehead as the measurement site because the brain tissue under the forehead is a vital CBT organ whose structure hardly changes with movement. The sternum can also be considered as a measurement site. However, the effect of the motion on the contact surface of the upper abdomen with the probe should be considered.

Therefore, the forehead was used as the measurement site in exercise-related experiments in subsequent human experiments. For sauna and heat therapy, the sternum was chosen as the safe temperature measurement site because the upper abdomen was smoother and unaffected by movement.

In addition, the simulation of the probe morphology can be further utilized for wearable improvements. Appropriate flattening and enlargement of the internal aluminum probe radius can yield better results and reach thermal equilibrium more quickly. It is worth mentioning that a balance needs to be struck between radius expansion and wearability, as a probe radius that is too large can lead to forehead discomfort and higher power consumption. In cases where high-precision measurements are required, the radius of the aluminum housing of the probe can be expanded, sacrificing some of the wearability, whereas a small-radius probe can be used appropriately for extended use, focusing on capturing the degree of temperature change rather than measurement accuracy.

### 4.2. Simulator Experiments

The simulator experiments showed a significant increase in water temperature during the second stage. The change in water temperature may be caused by hot air, which continuously heats the aluminum tray and indirectly heats the water in the narrow tank. The prototype probe accurately recorded the temperature changes under a strong hot wind.

Compared with the room-temperature environment, where the temperature difference between the inside and outside of the probe was tremendous, the absolute value of V_Input_ was greater in a high-temperature environment. This may be because of the higher wind speed around the radiator, which brings more heat to the interior by thermal convection, and the Peltier element transmitting more heat to counter the effect of thermal convection during operation.

### 4.3. Simultaneous Measurement of the Prototype and CTM-210 in Ergometer Exercise at Room Temperature

The prototype exhibited a response rate comparable to or better than that of a conventional ZHF probe to temperature changes induced by motion at room temperature, confirming the sensitivity of the measurements.

The dual-heat flux method of Ming et al. [28] removes the heater and uses changes in the heat flux to monitor the core body temperature. In a similar ergometer exercise experiment, the time delays of the two probes (fit and standard types) were 0 and 3 min, respectively, relative to the CTM-210 probe, which was also the reference. With the results of Bland–Altman analysis performed with the CTM-210 probe, the mean difference was −0.07 °C and −0.07 °C, and the 95% confidence intervals of the differences were (0.26 °C, −0.40 °C) and (0.12 °C, −0.27 °C), respectively, for the fit and standard type probes.

In comparison, our prototype probe had a lower time delay (−1.2 min–0 min) and higher accuracy (mean difference = 0.01 °C, 95% confidence interval of the differences = (0.21 °C, −0.23 °C) than the fit type relative to the two probes of the DHF method.

### 4.4. Simultaneous Measurement of the Prototype and CTM-210 in the Domed Sauna Experiment

The advantage of the prototype probe with Peltier elements is clearly demonstrated by the results of the domed sauna experiment, as shown in Figure 9. In all subjects, the temperatures recorded by the prototype probe were not affected by the air temperature rise in Phase B, except in Subject (c), while the temperatures recorded by the CTM were largely affected by air temperature; even in Subject (c), the effect of air temperature rise was significantly smaller than that in the CTM. It can be observed that the temperature trends obtained by the prototype probe were almost parallel to the trends of head temperatures. These facts suggest that the Peltier element can work satisfactorily to maintain a zero-heat-flux condition, even when the air temperature is elevated above the body core temperature.

In Phase A, the results of T_Head_ differed from those obtained from the abdominal measurements (T_Proto_ and T_CTM_). Owing to the different measurement sites, the difference between the abdominal temperatures and T_Head_ could not be used as an indicator of the accuracy of the measured temperature. However, it can be used as a reference value because the change in the CBT can be estimated as the change in T_Head_.

In addition, some results show that T_Proto_ differs from T_CTM_ at the end of Phase A. We assumed that this difference was caused by differences in the organs below the probe. The organs beneath the probe may vary with the location of the probe attachment, thus causing some differences in the temperature in the abdominal measurements. Studies [29] have demonstrated that the deep temperature measured in different organs varies, with the stomach often having a higher temperature than the liver. Although both probes were fixed at the same height in the upper abdomen, the lower part of the prototype probe was closer to the stomach, whereas the lower part of the ZHF probe tended to be closer to the liver. This may be one of the reasons for this temperature difference; in six of the seven subjects, T_Proto_ was virtually unaffected by changes in T_Air_ compared with the drastically changing T_CTM_. This difference was also consistent with the results of the finite element analysis, which confirmed the validity of the Peltier element.

We assumed that in Subject (c), the deep temperature below the probe was influenced by the blood from the surrounding body surface, leading to a change in T_Proto._ Therefore, owing to body–shell tissue thickness, thermal conductivity, and the effect of subcutaneous peripheral blood, we recommend using the forehead as a site for CBT measurements in a practical sauna or sports workplace setting as opposed to the abdominal solar plexus.

Our PID control system design accomplishes the desired control objectives in terms of input voltage variation. The PID system provides more accurate temperature control with less temperature fluctuation and helps reduce power wastage due to overshooting, which improves wearability.

In the second phase, the maximum average voltage achieved by the Peltier module under cooled conditions (0.31 V) was lower than the maximum voltage in the water bath test (approximately 1.07 V). This difference may be due to the higher hot-air wind speed in the simulator experiment, which is a strong convection, whereas in the domed sauna, there was almost natural thermal convection. At higher wind speeds and larger temperature, differences between the inside and outside of the probe, V_Input_, are correspondingly larger.

### 4.5. Limitations

The numerical calculation uses a homogeneous domain to simplify the model by treating the skin, subcutaneous fat, muscle, bones, and other tissues separated between the probe and the organ in the same area and assigning the same thermal conductivity to focus on the performance of the probe in establishing thermal equilibrium at different external temperatures. The actual measurement process is affected by the tissue composition, skin heat dissipation, metabolic efficiency, etc., below the measurement site, and the actual results may be inaccurate from the numerical calculation. Therefore, the possible effects of perfusion are not reflected in the simulation.

For the domed sauna experiment, the core body temperature from the forehead was recorded as a reference value. Brain temperatures differed from those of internal organs; however, for the subject’s safety, the heads were not placed in the sauna dome. Consider using a capsule thermometer that can be referenced to the esophageal temperature.

In addition, we found that, in practice, the exact location and depth of the specific measured CBT is difficult to achieve owing to the different thickness and composition of the body surface tissue.

The measurement time in a high-temperature environment was only 20 min, owing to the safety considerations of the subjects. The accuracy of human measurements over a longer period is not yet known.

In addition, a domed sauna provides a temperature increase mainly due to thermal radiation without the occurrence of strong convection conditions. In practical applications, if the probe is placed on the forehead and exposed to outside air, a strong convection environment may arise because of motion and other actions. This experiment initially verified its performance in a high-temperature natural-convection environment. However, the performance of this device on the human body in a strong convection environment requires further exploration.

### 4.6. Future Work

We redesigned the structure of the probe based on the co-author’s research and achieved the first measurement of this probe on a human body at high temperatures. Simulation, simulator, and simultaneous measurement experiments demonstrated that our probe can maintain thermal equilibrium and measurement accuracy at high temperatures comparable to conventional ZHF probes. We have a higher measurement accuracy (mean difference −0.01 °C, 95% limits of agreement −0.23 to 0.21 °C) than tympanometry [10] (0.22 °C, −0.44 to 1.30 °C) and wrist thermometers [16] (1.51 °C, −1.34 to 4.35 °C). We also have a more stable measurement site and measurement time than ingestible capsule thermometers, which make it difficult to control the measurement site and time.

Building on the current design, future studies should focus on improving the wearability of the probe.

The current design may not be sufficient for extended measurements over 24 h because strong winds and lower outside temperatures can cause a greater voltage to be applied to the Peltier when achieving temperature equilibrium, thereby increasing the power consumption. A combination of a heater and Peltier element was considered to reduce the power consumption of the prototype probe. The Peltier element can maintain thermal equilibrium at high temperatures; however, its conversion efficiency is lower than that of the heater at room temperature. More power can be utilized according to the Peltier element operation to cool when the ambient temperature is higher than the core body temperature, and a heater is used to heat the inside of the probe at room temperature.

Regarding the current design, there have been many requests to improve the wearability of the probe. The current size is too large for a head-mounted device and it has harder edges that do not fit very well to the forehead. In the simulator experiment, the current heat sink was sufficient to support heat dissipation under strong convection conditions of up to 65 °C. However, their actual use may not reach extreme environmental conditions. Therefore, the size of the radiator can be reduced appropriately. In addition, it is possible to improve the edge fit and reduce the overall size of the probe appropriately by replacing it with a softer and better insulating material.

In addition, dual-heat-flux thermometers may also have some promise for wearable thermometers if they do not require excessive accuracy but focus only on capturing abnormal changes in the core body temperature. Although the principle of dual-heat-flux thermometry was recognized theoretically [30], little research has been conducted until recently, where it was introduced in wearable thermometers [2]. Owing to the advantages of low-power operation and the capability to operate in high-temperature environments, it warrants further research.

## 5. Conclusions

In this study, we designed and experimentally verified a ZHF probe that could be monitored in high-temperature environments. Experiments showed that the prototype could accurately track rapid changes in CBT at room temperature and measured temperature in high-temperature environments, which conventional ZHF methods cannot.

Because the improved ZHF method is available even in high-temperature environments, it is expected to aid athletes, outdoor workers, firefighters, and others in monitoring their body temperature in high-temperature environments.

## Figures and Tables

**Table 1:** Assumed values of the probe materials.

Component	Thermal Conductivity W/(m·K)	Density kg/m^3^	Specific Heat J/(kg·K)	Electrical Conductivity S/m	Seebeck Coefficient V/K
Skin	0.48	1.1 × 10^3^	3.4 × 10^3^	/	/
Aluminum	240	2.7 × 10^3^	900	/	/
Closed-cell foam	0.04	210	16	/	/
Foam	0.04	24	2.2	/	/
Alumina	27	3.92 × 10^3^	900	/	/
Copper	400	9.0 × 10^3^	390	6 × 10^7^	/
P-Bi2Te3	1.6	7.7 × 10^3^	150	8.7 × 10^4^	2.1 × 10^−4^
N-Bi2Te4	1.6	7.7 × 10^3^	150	8.7 × 10^4^	−2.1 × 10^−4^

**Table 2:** Assumed values in the finite element analysis.

Quantity	Notation	Assumed Value
External temperature	T_EXT_	25/42 °C
Core body temperature	T_CBT_	37 °C, 0≤t≤ 2000 s 38 °C, 2000<t≤ 3500 s
Convection heat transfer coefficient (free convection, gases)	h	5 W/(m^2^·K)
Proportional gain	K_p_	3
Integral gain	K_i_	1
Derivative gain	K_d_	1

## Data Availability

Not applicable.
